# Association of frailty and chronic limb-threatening ischemia in patients on maintenance hemodialysis: a prospective cohort study

**DOI:** 10.18632/aging.206178

**Published:** 2024-12-31

**Authors:** Mu-Yang Hsieh, Chien-Ming Luo, Chi-Hong Cheng, Li-Pei Dai, Chiu-Hui Chen, Shao-Yuan Chuang, Chung-Wei Yang, Chih-Cheng Wu

**Affiliations:** 1 College of Medicine, National Taiwan University, Taipei, Taiwan; 2 Department of Medicine, Cardiology Division, National Taiwan University Hospital, Hsin-Chu Hospital, Hsin-Chu, Taiwan; 3 Department of Surgery, Cardiovascular Division, National Taiwan University Hospital, Hsin-Chu Hospital, Hsin-Chu, Taiwan; 4 Ansn Clinic, Hsin-Chu, Taiwan; 5 Department of Internal Medicine, Catholic Mercy Hospital, Hsin-Chu, Taiwan; 6 Hemodialysis Center, National Taiwan University Hospital, Hsin-Chu Hospital, Hsin-Chu, Taiwan; 7 Institute of Population Health Science, National Health Research Institutes, Zhunan, Taiwan; 8 Quality Control Center, National Taiwan University Hospital, Hsin-Chu Hospital, Hsin-Chu, Taiwan; 9 Institute of Biomedical Engineering, National Tsing-Hua University, Hsin-Chu, Taiwan; 10 Institute of Cellular and System Medicine, National Health Research Institutes, Zhunan, Taiwan

**Keywords:** follow-up, frailty, major adverse limb events, peripheral artery disease, prospective cohort study

## Abstract

Chronic limb-threatening ischemia (CLTI) is a prevalent yet unpredictable complication among patients undergoing hemodialysis, and frailty is linked to adverse outcomes in this population. This study examined the influence of clinical factors on vascular events in patients undergoing hemodialysis. This multicenter prospective cohort study included patients undergoing maintenance hemodialysis since January 2008. The initial cohort consisted of 1,136 patients, 828 of whom successfully underwent a frailty test. CLTI events were recorded at 3-month intervals until December 31, 2022. The mean patient age was 67 years, and 48% were female. Overall, 34% of participants were frail, 38% pre-frail, and 28% not frail. Frailty phenotype was associated with age, female sex, low educational level, diabetes mellitus, and history of stroke. During a median follow-up of 1461 days, 104 patients experienced CLTI events (not frail, 6.5%; pre-frail, 11%; frail, 20%; *P* < 0.001). Frail patients had a higher risk of CLTI than those who were non-frail (hazard ratio (HR) 3.94; 95% confidence interval (CI) 2.22–6.99; *P* < 0.001). After multivariable adjustment for age and comorbidities, frailty remained significantly associated with CLTI (HR 3.26; 95% CI 1.76–5.85; *P* < 0.001). Conclusively, these findings highlight the risk of CLTI in frail patients undergoing hemodialysis.

## INTRODUCTION

Patients with end-stage renal disease (ESRD) have a higher prevalence of peripheral arterial disease (PAD) than the general population [1, 2]. PAD can manifest with mild or no symptoms, intermittent claudication, or chronic limb-threatening ischemia (CLTI) [3]. CLTI is a late stage of PAD, defined as limb pain that occurs at rest or impending limb loss caused by severe compromise of blood flow to the affected extremity [3]. Patients with CLTI have the worst PAD outcomes; however, the progression to CLTI is often variable and unpredictable [4]. Thus, approaches to maximize early detection and management of CLTI have been an issue in managing patients undergoing hemodialysis.

Frailty is the increased susceptibility to stressors resulting from impairments across physiological, psychological, and socioeconomic domains [5]. It is characterized by muscle weakness, inadequate nutrition, reduced physical activity, and comorbidities, differentiating it from traditional risk factors [6]. Observational studies have established a significant correlation between frailty and various outcomes such as mortality, cognitive dysfunction, quality of life, and cardiovascular disease in the dialysis population [7–14]. Frailty is highly prevalent in the hemodialysis population, with an estimated prevalence of 29–46% [10, 15, 16]. Recent meta-analyses also noted that the prevalence of frailty was approximately 50% in patients with lower extremity PAD [17–21]. PAD may also be a contributing factor to the diagnosis of frailty [5, 22]. The relationship between PAD and frailty in patients undergoing dialysis remains to be comprehensively investigated. Prospective data are needed to clarify the causal relationship and prognostic value of frailty in adverse events among patients undergoing hemodialysis [23, 24].

We hypothesize that frailty might result in delayed recognition and treatment of PAD and lead to CLTI [23]. The Hsinchu Vascular Access (Hsinchu VA) study was a prospective cohort study that investigated the relationship between clinical factors and incident cardiovascular diseases in patients undergoing hemodialysis [24]. In a substantial proportion of these patients, performance-based frailty status was assessed at enrollment, and PAD events were prospectively documented during the follow-up period [24]. We conducted a pre-planned secondary analysis of the Hsinchu VA study to test the hypothesis that frail patients would have a higher incidence of CLTI than non-frail patients and examine the association of frailty with other major adverse limb events (MALE).

## RESULTS

### Study participants

This current analysis included 828 patients who had completed the frailty assessment from the original cohort of 1,136 patients. Baseline characteristics were similar to those in patients undergoing hemodialysis in a recent nationwide registry in Taiwan (Supplementary Table 1).

Table 1 shows the baseline characteristics and comorbidities of the study participants. The mean age (standard deviation) of the study participants was 67 (14) years, and 395 (48%) were female. The median duration of hemodialysis was 3.7 (interquartile range (IQR), 0.6–5.6) years, and the median duration of follow-up was 4 (IQR, 2.8–5.0) years. The study participants had various comorbidities: 724 (87%) had hypertension, 436 (53%) had diabetes mellitus (DM), 202 (24%) had hyperlipidemia, and 119 (14%) were smokers. Some patients had pre-existing vascular diseases at enrollment, including 217 (26%) with coronary artery disease (CAD), 73 (8.8%) with PAD, and 76 (9.2%) with cerebrovascular accident (CVA).

**Table 1:** Baseline characteristics of the study participants stratified by frailty status.

**Variable**	**Overall, *N* = 828^*a*^**	**Not Frail, *N* = 231^*a*^**	**Pre-Frail, *N* = 317^*a*^**	**Frail, *N* = 280^*a*^**	***P*-value^*b*^**
**Demographic and socioeconomic factors**					
Age (years)	67 (14)	62 (13)	65 (15)	71 (12)	<0.001
Age >65 years	432 (52%)	88 (38%)	149 (47%)	195 (70%)	<0.001
Female sex	395 (48%)	91 (39%)	149 (47%)	155 (55%)	0.001
BMI (kg/m^2^)	22.9 (4.2)	23.1 (4.0)	22.9 (4.3)	22.7 (4.4)	0.40
Education >6 years	514 (62%)	161 (70%)	211 (67%)	142 (51%)	<0.001
Married	636 (77%)	175 (76%)	255 (80%)	206 (74%)	0.13
Current smoker	119 (14%)	41 (18%)	44 (14%)	34 (12%)	0.20
**Comorbidities**					
Hypertension	724 (87%)	204 (88%)	278 (88%)	242 (86%)	0.80
DM	436 (53%)	97 (42%)	158 (50%)	181 (65%)	<0.001
Hyperlipidemia	202 (24%)	63 (27%)	79 (25%)	60 (21%)	0.30
CAD	217 (26%)	47 (20%)	84 (26%)	86 (31%)	0.03
PAD	73 (8.8%)	14 (6.1%)	25 (7.9%)	34 (12%)	0.04
CVA or ICH	76 (9.2%)	9 (3.9%)	29 (9.1%)	38 (14%)	<0.001
CHF	101 (12%)	28 (12%)	33 (10%)	40 (14%)	0.35
AF	114 (14%)	23 (10.0%)	41 (13%)	50 (18%)	0.03
Liver disease	90 (11%)	21 (9.1%)	43 (14%)	26 (9.3%)	0.15
COPD	19 (2.3%)	9 (3.9%)	6 (1.9%)	4 (1.4%)	0.15
Cancer	72 (8.7%)	12 (5.2%)	35 (11%)	25 (8.9%)	0.06
**Dialysis factors**					
Dialysis vintage (years)	3.7 (0.6, 5.6)	4.0 (0.8, 6.8)	3.4 (0.6, 5.6)	3.2 (0.6, 5.2)	0.14
Fluid removal (%)	3.57 (1.42)	3.74 (1.43)	3.64 (1.33)	3.34 (1.47)	0.007
Dialysis frequency <3 per week	56 (6.8%)	14 (6.1%)	21 (6.6%)	21 (7.5%)	0.80
Albumin (g/dL)	3.83 (0.34)	3.92 (0.30)	3.84 (0.35)	3.75 (0.32)	<0.001
Kt/V (Daugirdas)	1.69 (0.29)	1.71 (0.31)	1.68 (0.27)	1.69 (0.30)	0.80
Hb (g/dL)	10.80 (1.37)	11.01 (1.43)	10.78 (1.37)	10.65 (1.30)	0.02
**Medications**					
Antiplatelet	212 (26%)	56 (24%)	79 (25%)	77 (28%)	0.70
Warfarin	11 (1.3%)	2 (0.9%)	3 (0.9%)	6 (2.1%)	0.40
Beta-blocker	145 (18%)	48 (21%)	54 (17%)	43 (15%)	0.30
RAAS inhibitors	133 (16%)	39 (17%)	58 (18%)	36 (13%)	0.20
Statin	123 (15%)	28 (12%)	54 (17%)	41 (15%)	0.30

^*a*^Mean (SD); *n* (%); Median (IQR). ^*b*^Kruskal–Wallis rank sum test; Pearson’s Chi-squared test; Fisher’s exact test. Abbreviations: AF: atrial fibrillation; BMI: body mass index; CAD: coronary artery disease; CHF: congestive heart failure; COPD: chronic obstructive pulmonary disease; CVA: cerebrovascular accident; DM: diabetes mellitus; Hb: hemoglobin; ICH: intracerebral hemorrhage; IQR: interquartile range; PAD: peripheral arterial disease; RAAS: renin-angiotensin-aldosterone system; SD: standard deviation.

### Prevalence of and factors associated with frailty

As shown in Table 1, 280 (34%) participants were categorized as frail, 317 (38%) as pre-frail, and 231 (28%) as robust. Frailty was more prevalent in patients who were older, female, and with low education levels. DM and CVA were associated with frailty. After multivariate adjustment, older age (>65 years), female sex, DM, CVA, and serum albumin levels remained significantly associated with frailty (Supplementary Table 2).

### Follow-up and events

**Figure 1:**
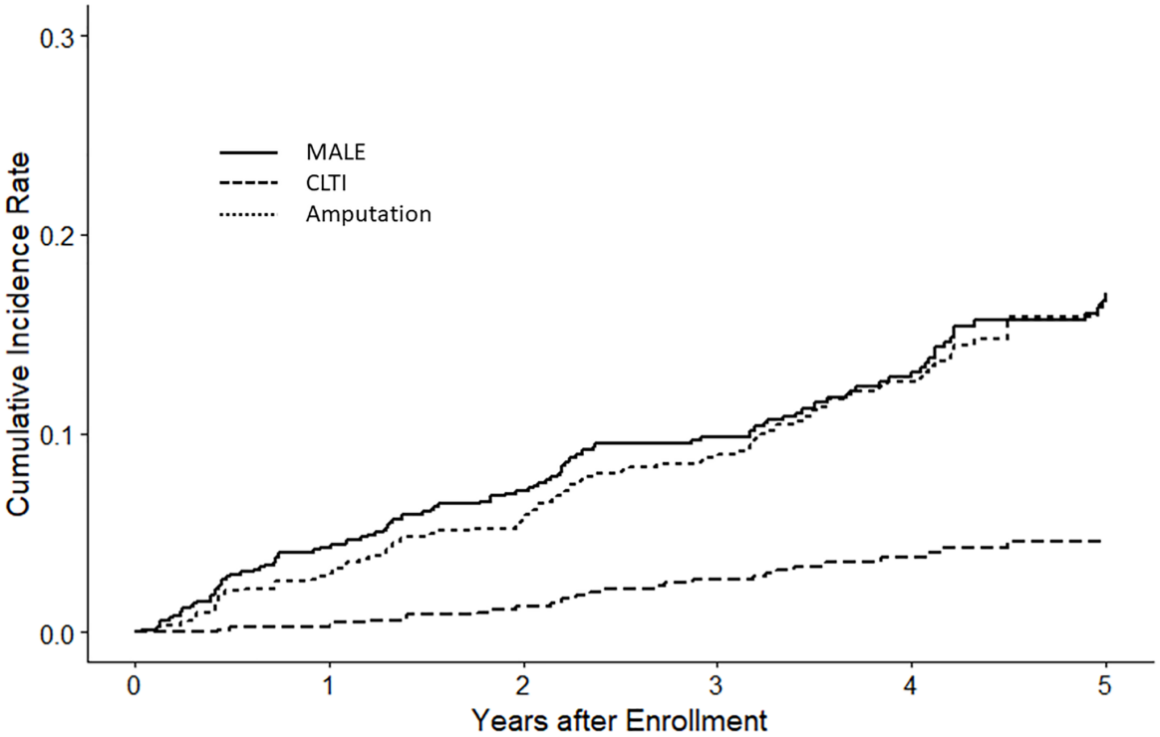
**Kaplan–Meier plot of lower limb vascular events.** The figure presents lower limb vascular events within the entire study cohort, encompassing major adverse limb events (MALE), chronic limb-threatening ischemia (CLTI), and amputations.

**Figure 2:**
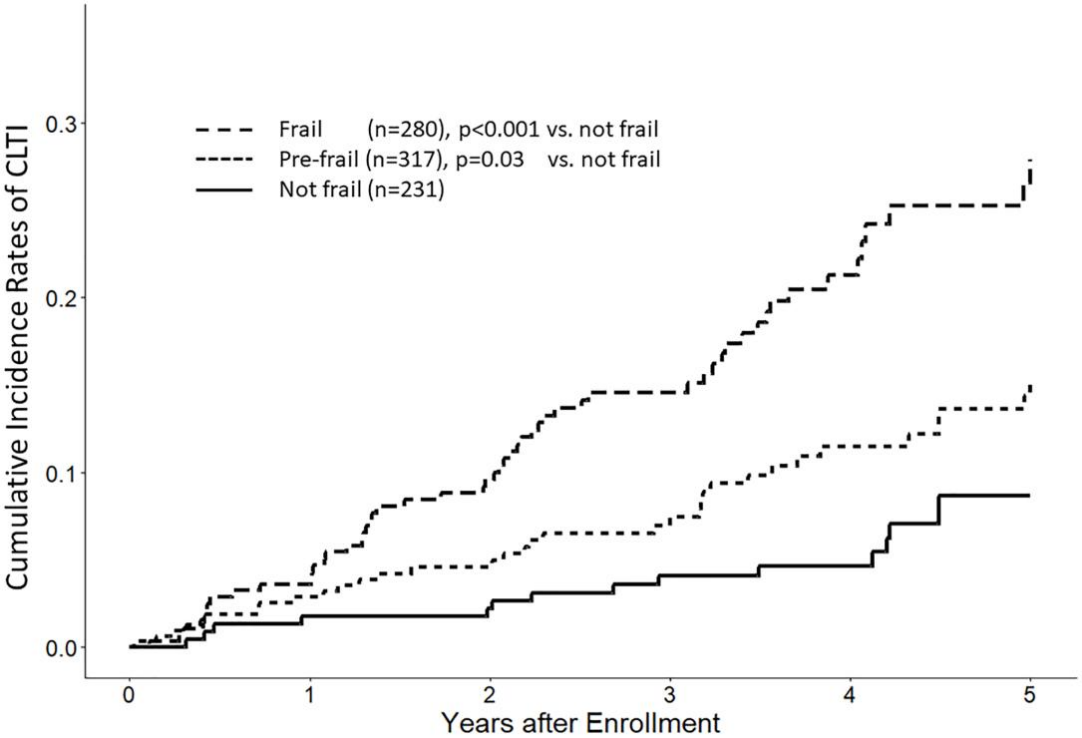
**Kaplan–Meier plot of critical limb ischemia events by frailty status.** This figure depicts the time to the occurrence of the first chronic limb-threatening ischemia (CLTI) event, categorized based on frailty status.

The median follow-up was 1,461 (IQR, 1,016–1,826) days. During the follow-up period, 4 (0.5%) patients received renal transplantation, 106 (13%) were transferred to non-study facilities, and 231 (28%) died (shown in Supplementary Figure 1). During the follow-up period, CLTI occurred in 104 patients (13%), 28 (3%) underwent amputation, and 101 (12%) underwent intervention. MALE occurred in 108 (13%) patients (shown in Figure 1). The Kaplan–Meier analysis demonstrated a graded relationship between CLTI events and frailty status (not frail, 6.5%; pre-frail, 11%; frail, 20%; *P* < 0.001) (shown in Figure 2 and Supplementary Table 3). There was also a graded effect between mortality and frailty status (not frail, 13%; pre-frail, 27%; frail, 41%; Supplementary Table 3).

### Cox regression analyses

Univariate Cox proportional hazards regression analysis showed that age, smoking, comorbidities (DM, hyperlipidemia, CAD, CVA, and atrial fibrillation), antiplatelet and statin use, and frailty status were associated with an increased risk of CLTI (Table 2). Frail patients had a more than threefold higher risk of CLTI than those who were non-frail (hazard ratio (HR) 3.94; 95% confidence interval (CI) 2.22, 6.99;* P* < 0.001). DM (HR 4.43; 95% CI 2.72–7.22; *P* < 0.001) and frailty status (HR 3.94; 95% CI 2.22–6.99; *P* < 0.001) were the most significant factors associated with the occurrence of CLTI. After adjusting for age, BMI, educational level, smoking, DM, hypertension, hyperlipidemia, CAD, CVA or ICH, atrial fibrillation, Kt/V, and hemoglobin in the multivariate analysis, frailty status remained significantly associated with an increased risk of CLTI (HR 3.26; 95% CI 1.76–5.85; *P* < 0.001) (Table 2).

**Table 2:** Cox regression analysis for factors associated with chronic limb-threatening ischemia.

**Characteristic**	**Univariable**			**Multivariable**		
	**HR^*a*^**	**95% CI^*a*^**	***P*-value**	**HR^*a*^**	**95% CI^*a*^**	***P*-value**
**Frailty score**						
Not frail	—	—		—	—	
Pre-frail	1.95	1.06–3.58	0.03	1.9	1.02–3.57	0.04
Frail	3.94	2.22–6.99	<0.001	3.26	1.76–5.85	< 0.001
**Demographic and socioeconomic factors**						
Age >65 (years)	2.15	1.43–3.22	<0.001	1.62	1.05–2.62	0.03
Female sex	0.87	0.59–1.28	0.48			
**BMI groups (kg/m^2^)**						
<18.5	—	—		—	—	
18.5–24	2.72	1.09–6.78	0.03	1.77	0.69–4.52	0.2
24–27	2.59	0.99–6.83	0.05	1.28	0.46–3.51	0.6
>27	2.98	1.10–8.08	0.03	1.72	0.60–4.95	0.3
Education <6 years	1.76	1.20–2.58	0.004	1.36	0.89–2.08	0.2
Married	0.78	0.51–1.20	0.26			
Current smoker	1.97	1.26–3.07	0.003	2.12	1.33–3.40	0.002
**Comorbidities**						
DM	4.43	2.72–7.22	<0.001	3.24	1.83–5.74	< 0.001
HTN	0.55	0.29–1.02	0.06	1.21	0.59–2.47	0.6
Hyperlipidemia	2.32	1.57–3.42	<0.001	1.36	0.87–2.12	0.2
CAD	2.25	1.53–3.32	<0.001	0.95	0.61–1.50	0.8
CVA or ICH	2.4	1.44–3.99	<0.001	1.5	0.89–2.54	0.13
CHF	1.12	0.64–1.97	0.69			
Atrial fibrillation	2.61	1.70–4.01	<0.001	1.9	1.21–2.98	0.005
COPD	1.22	0.39–3.84	0.74			
**Dialysis factors**						
Dialysis vintage (years)	0.97	0.94–1.01	0.2			
Cholesterol (mg/dL)	1	0.99–1.00	0.34			
Albumin (g/dL)	1.05	0.58–1.90	0.86			
Kt/V (Daugirdas)	0.36	0.18–0.72	0.004	0.76	0.35–1.64	0.5
Hb (g/dL)	1.24	1.08–1.42	0.002	1.24	1.08–1.44	0.003
**Medications**						
Antiplatelet	3.05	2.07–4.48	<0.001	2.1	1.33–3.32	0.001
Warfarin	1.26	0.31–5.12	0.74			
Beta-blocker	1.22	0.76–1.97	0.41			
RAAS inhibitors	0.68	0.38–1.21	0.19			
Statin	1.91	1.22–3.00	0.005	1.11	0.69–1.81	0.7

^*a*^HR: Hazard Ratio; CI: Confidence Interval. Abbreviations: BMI: body mass index; CAD: coronary artery disease; CHF: congestive heart failure; COPD: chronic obstructive pulmonary disease; CVA: cerebrovascular accident; DM: diabetes mellitus; Hb: hemoglobin; HTN: hypertension; ICH: intracerebral hemorrhage; RAAS: renin-angiotensin-aldosterone system.

### Subgroup analysis

**Figure 3:**
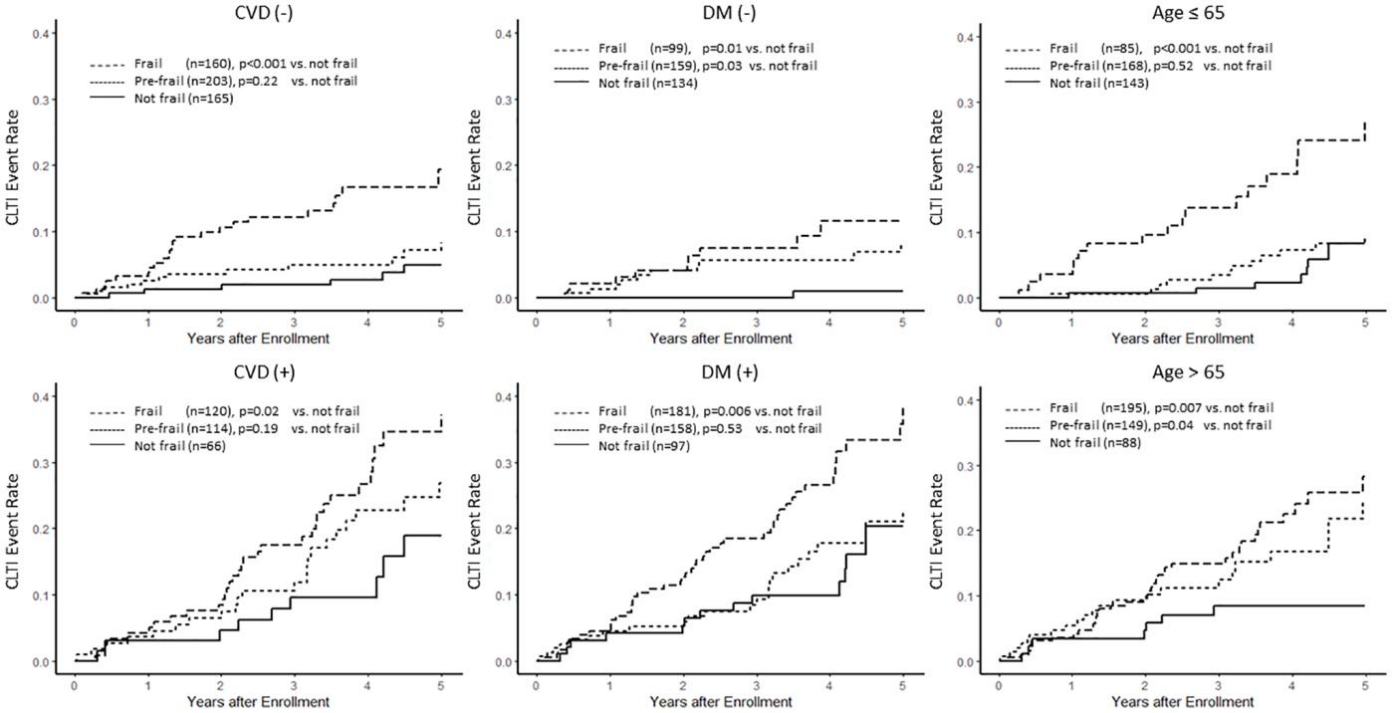
**Association between frailty and the risk of chronic limb-threatening ischemia (CLTI) by different subgroups.** This figure demonstrates the Kaplan–Meier curves of chronic limb-threatening ischemia (CLTI) stratified by frailty status in different subgroups, including cardiovascular diseases (CVD), diabetes mellitus (DM), and age.

After stratification by age of 65 years, DM, and pre-existing vascular diseases, the frailty status remained associated with CLTI events (Figure 3). Frail patients experienced a higher incidence of CLTI events compared to non-frail dialysis patients, with pre-frail patients exhibiting an intermediate incidence.

## DISCUSSION

The main finding of our study was that frailty was associated with the development of CLTI in patients undergoing hemodialysis. As hypothesized, both frail and pre-frail patients had a higher risk of CLTI events compared to non-frail patients, with a graded effect. Frail individuals exhibited the highest incidence of CLTI events, followed by pre-frail individuals, while non-frail individuals experienced the lowest CLTI events. After adjusting for potential covariates, frail patients exhibited a 3.26-fold increased risk of developing CLTI compared to non-frail patients. Consequently, frailty appears to serve as a predictive factor for CLTI in individuals undergoing hemodialysis.

Various frailty tests are currently available. We used the original performance-based measures proposed by Fried et al [25]. Although cut-offs for gait speed were modified in our study [22, 26], the frailty prevalence of 34% was consistent with that reported in the United States (29–34%) and Asian countries (35%) [15, 16, 22, 26]. Our data confirmed previous observations that frailty was common in the hemodialysis population and is consistent across different ethnic groups. Nevertheless, approximately 27% of hemodialysis patients (308 out of the total cohort of 1,136 patients) in the study did not undergo performance-based assessments, which could have led to an underestimation of the true prevalence of frailty.

In the general population, the prevalence of PAD is 5.6%, and that of CLTI is 1.3% among individuals who are more than 40 years old [27, 28]. The ESRD population has a much higher prevalence of PAD than the general population [2, 29]. For example, the Dialysis Outcomes and Practice Patterns Study reported an 11.5–37.8% prevalence rate of PAD with significant geographic variations [29]. In our study, at the time of patient enrollment, 8.8% of patients had a diagnosis of PAD. This prevalence appears lower than that reported in previous studies, which may be attributed to several factors. First, some patients were in the early stages of initiating hemodialysis, and ankle-brachial index measurements had not yet been conducted. Second, some patients may have been asymptomatic and, consequently, had not received a PAD diagnosis. Nevertheless, we observed a significantly higher CLTI incidence than the general population during the follow-up period. In the general population, the annual incidence of PAD was 0.2/100 person-years (PY), and the incidence of CLTI was approximately 0.02/100 PY [17]. In the hemodialysis population, the incidence of PAD or CLTI has rarely been reported in prospective studies [30, 31]. In our cohort, patients undergoing hemodialysis exhibited a CLTI incidence rate of 3.3 per 100 PY, significantly higher than that reported in the general population [27]. Our findings were compatible with previous evidence that ESRD patients have an eight to 20-fold risk of mortality from cardiovascular events [32–34]. This trend of increased cardiovascular events among ESRD patients has also been observed in the Asian population [35]. Various investigators have reported an increased risk of myocardial infarction, ischemic stroke, acute limb ischemia, and deep vein thrombosis for ESRD patients [29, 35–39]. ESRD patients have increased susceptibility to cardiovascular disease and dysfunction and often have elevated cardiac troponin serum concentrations due to chronic heart muscle injury. Our findings suggest that beyond traditional biomarkers, frailty can be used as an additional clinical risk stratification marker.

To date, no prospective study has examined the relationship between frailty and CLTI or MALE in patients undergoing hemodialysis. For the first time, our cohort demonstrated that assessing frailty helped identify the risk of CLTI events in patients undergoing hemodialysis. The risk of CLTI associated with frailty was more prominent than that associated with most conventional vascular risk factors, except for DM.

As demonstrated in previous studies, DM, aging, and pre-existing vascular disease are well-established risk factors for CTLI [31]. In this study, frailty status remained a significant predictor of CLTI even after adjusting for these factors in the multivariate analysis. Specifically, frail patients had a more than threefold increased risk of CLTI compared to non-frail patients, with pre-frail patients showing an intermediate risk. This graded relationship between frailty and CLTI incidence persisted across various subgroups, including those stratified by age (65 years), presence of DM, and pre-existing vascular diseases. By adjusting for these variables in the multivariate analysis and subgroup analysis, the study robustly demonstrates that frailty independently contributes to the risk of CLTI. This highlights the importance of considering frailty in the management and prognosis of patients undergoing hemodialysis, as it significantly impacts the risk of severe vascular complications regardless of traditional risk factors.

Numerous studies have presented the possibility of an epidemiological and pathophysiological relationship between PAD and frailty. This connection may arise from factors such as aging, shared risk factors, and chronic inflammation, leading to an overall decline in physical function or the development of microvascular and macrovascular complications due to chronic vascular inflammation [17, 40–42]. In our study, we focused on CLTI. The most intuitive link between frailty and CLTI is their shared risk factors. Various mechanisms may explain this association. Frailty and CLTI may share common biological pathways. The pathophysiology of CLTI involves not only obstructive macrovascular disease but also microvascular changes secondary to inflammation, a hypercoagulable state, impaired angiogenesis, and vasomotor paralysis [43]. Previous studies have demonstrated that frailty is associated with increased coagulation markers and the risk of thromboembolism in older patients [44, 45]. Scientific evidence also suggests that inflammation (interleukin-6 and C-reactive protein), oxidative stress, and endothelial dysfunction play important roles in the pathogenesis of frailty [46–48].

Malnutrition and inflammation are often linked with frailty and may contribute to the progression of chronic limb-threatening ischemia (CLTI). The frailty status, frequently associated with malnutrition or immunocompromised states, is part of the malnutrition-inflammation-atherosclerosis (MIA) syndrome, which has been proposed as a cause of increased mortality in ESRD patients [49]. CLTI, as a manifestation of advanced atherosclerotic vascular disease, could be influenced by these factors. In our study, although low serum albumin levels were associated with frailty, they were not correlated with CLTI in our analysis. This finding aligns with the observations by Koch et al., who reported that nutritional status alone does not predict cardiovascular mortality [50]. Similarly, Struijk et al. indicated that serum albumin levels primarily reflect the presence of systemic diseases rather than being a direct predictor of cardiovascular outcomes in dialysis patients. Additionally, our study did not perform comprehensive nutritional assessments or measure inflammatory biomarkers, limiting our ability to fully evaluate the impact of malnutrition and inflammation on CLTI. The results suggest that while frailty is a significant predictor of CLTI, its association with malnutrition and inflammation requires further investigation.

We also found that frailty was associated with a low educational level; the educational level has been identified as a critical component in improving PAD outcomes [51]. Low levels of education can create significant barriers to effective foot care and access to health resources [52]. Finally, reduced physical activity and walking speed are the hallmarks of frailty, as decreased physical activity is associated with atherosclerotic vascular disease, and exercise has been shown to be beneficial for patients with PAD [53, 54]. Therefore, the correlation is biologically plausible because the beneficial effects of physical activity on cardiovascular risk function may be less in frail patients. Exertional leg symptoms may be masked due to inactivity or slow pace in frail patients in whom PAD was not recognized until CLTI occurred.

This study has several strengths. The prospective design ensured that data on lower limb vascular events were collected after frailty assessment with a comprehensive collection of covariates. These approaches enabled us to analyze the causal relationship between frailty and limb events in a temporal sequence to avoid reverse causation. To reduce the bias arising from outcome misclassification, we used medical records containing imaging information and consulted the treating physicians as required. The frailty assessment was based on performance-based measurements rather than questionnaires, which enabled direct comparison with other studies.

However, it is important to acknowledge some limitations in this study. First, the presence of unmeasured confounding variables cannot be completely ruled out. Second, certain criteria used for assessing frailty were adapted to the aging population in Taiwan, potentially limiting the comparability of prevalence statistics with other populations. Third, the reliance on chart records to define PAD outcomes may have resulted in the underreporting of lower limb events. Additionally, the lack of comprehensive ankle-brachial index screening at enrollment could have made PAD unrecognized. Finally, a significant proportion of patients could not undergo frailty assessments, and their exclusion may have limited the generalizability of our study findings to this subset of the population.

PAD is common in patients with kidney failure and often presents as the most severe form of CLTI. Despite its high prevalence, the diagnosis of PAD in patients with kidney failure is challenging because patients often do not present with the classic symptoms of claudication [55, 56]. Incorporation of a frailty assessment, a relatively convenient and objective tool, may help stratify the risk of MALE. The National Kidney Foundation Kidney Disease Outcomes Quality Initiative guidelines on cardiovascular disease in patients undergoing dialysis recommend that all patients should be evaluated for PAD when they initiate dialysis, using a vascular physical examination, duplex ultrasound, or invasive testing as clinically indicated [57]. However, given the increasing pressure on healthcare resources, our results suggest that frail patients may represent a high-risk subgroup that may benefit from PAD screening. Considering the negative consequences of MALE, the assessment of frailty may help improve risk stratification.

In conclusion, patients with frailty who undergo hemodialysis have a high prevalence of PAD and are at an increased risk of developing CLTI. Our study found that frailty was independently associated with worse CLTI outcomes, even after adjusting for relevant risk factors. This observation suggests that evaluating the frailty status can be valuable in identifying individuals with an increased risk of CLTI and its associated complications. Further research is necessary to determine whether interventions aimed at mitigating the negative effects of frailty can improve limb outcomes in patients with CLTI.

## METHODS

### Study design and participants

The Hsinchu VA study was a multicenter prospective study investigating the impact of clinical factors on vascular events in patients undergoing maintenance hemodialysis (ClinicalTrials.gov identifier: NCT04692636). The original study cohort enrolled 1,136 patients on maintenance hemodialysis as of January 1, 2018, from 12 hemodialysis centers in Hsin-Chu, Taiwan, including 4 hospital-based dialysis centers and 8 local dialysis clinics. The inclusion criteria were age 18–90 years, receiving maintenance dialysis for more than 6 months, and not being hospitalized in the previous 3 months. Patients from the original cohort who completed the performance-based frailty evaluation were included in this analysis (shown in Supplementary Figure 1). The study was approved by the institutional review board (106-033-E), and all patients provided informed written consent before enrollment.

### Collection of data and follow-up of outcomes

Information on patient demographics, comorbidities, medications, lower limb vascular duplexes, hospitalization records, angiography, dialysis-related data, and laboratory results was obtained from the medical records of the dialysis centers. Trained study coordinators collected this information at baseline and every 3 months thereafter, including questionnaires, medical records, imaging studies, and surgery reports. The data obtained by the study coordinators were coded after review by two investigators (MYH and CCW), and discussions with primary physicians were conducted as needed. Patients were followed up until December 31, 2022, and censored in case of death, kidney transplant, or transfer to a non-study center. The primary outcome of Hsinchu VA study was vascular access thrombotic event. PAD-related events including CLTI, MALE, and amputation were secondary outcomes. The primary purpose of this post-hoc analysis was to report the incidence and associated factors of CLTI in the study cohort.

### Diagnosis of PAD and adverse limb events

At enrollment, the history of PAD was based on diagnosis from medical records or endovascular or surgical interventions (including amputation) for PAD. After enrollment, major adverse limb events were documented by interval questionnaire and review of medical records every 3 months. The diagnosis was confirmed by two investigators (MYH and CCW) based on the following criteria: abnormal duplex waveform or stenosis >50% on angiography imaging, endovascular or surgical revascularization, or PAD-related amputation or death. MALE encompassed vascular amputation and repeated vascular interventions. CLTI was defined as the presence of ischemic rest pain (Rutherford stage 4), ulcers, or gangrene (Rutherford stages 5 or 6), whether hospitalization or intervention was undertaken or not. Major vascular amputation was defined as an amputation resulting from a vascular event above the forefoot. Peripheral vascular interventions were classified as interventions for PAD, including peripheral angioplasty or vascular surgery, regardless of the Rutherford stage. The methodology for ascertaining the diagnosis of PAD has been previously described [23].

### Assessment of frailty

We assessed the Fried frailty phenotype at baseline and 2 years after enrollment [8, 58]. Unintentional weight loss was defined as a loss of more than 3 kg or 5% of the patient’s body weight within the previous year. Exhaustion was determined by a positive response to the questions on the vitality scale of the 36-Item Short Form Health Survey [59]. Low physical activity was defined using the Taiwanese version of the International Physical Activity Questionnaire-Short Form, which calculated calorie consumption and was based on the lowest 20% for each sex (<685 kcal/week in males and <420 kcal/week in females) [60]. We conducted grip strength tests on each hand three times using a handheld dynamometer (JAMAR; Lafayette Instrument Company, Lafayette, IN, USA) and recorded the average of the strongest hand. Cut-off values were defined according to the sex and body mass index (BMI) criteria provided in the study by Fried et al [25]. We measured gait speed by having participants walk at their usual pace over a 5-meter course and recording the fastest speed of the two trials. The Taiwanese-modified Fried criteria were used to determine the cut-off points [22]. Each component of the frailty phenotype was scored as 1 if present and 0 if absent, resulting in a frailty score ranging from 0 to 5. The participants were categorized into three states based on their scores: frail (3–5 points), pre-frail (1–2 points), and non-frail (0 points). The three frailty states were based on a study by Fried et al. and have been shown to be associated with mortality in hemodialysis populations [8, 25].

### Covariates

Demographic, socioeconomic, and smoking status data were collected by reviewing patient medical records. BMI was calculated by dividing weight in kilograms by the square of height in meters. BMI values were classified into four categories: underweight (<18.5 kg/m^2^), normal weight (18.5–24 kg/m^2^), overweight (24–27 kg/m^2^), and obese (>27 kg/m^2^) according to the criteria suggested for the Asian population [61, 62]. Comorbidities, such as CAD (including angina pectoris, myocardial infarction, positive non-invasive or invasive test results, or those requiring interventions), PAD, CVA, congestive heart failure, diabetes mellitus (DM), hypertension, chronic liver disease, atrial fibrillation, chronic obstructive pulmonary disease, and cancer were recorded according to the diagnosis at the dialysis units, documented by the patient’s primary nephrologist.

### Statistical analysis

Baseline characteristics were compared according to frailty status using analysis of variance for normally distributed continuous variables, the Kruskal–Wallis test for non-normally distributed continuous variables, and the χ^2^ test for categorical variables. We used the Shapiro-Wilk test to examine whether the continuous variable was normally distributed. Logistic regression analysis was performed to determine the factors associated with baseline frailty status, which were categorized into binary variables. Outcomes, including PAD events, were tabulated, stratified by frailty status, and assessed using the Kaplan–Meier method with a log-rank test. For each approach, data were censored in case of death, kidney transplantation, transfer to peritoneal dialysis, or loss to follow-up. Only baseline variables were used as exposures for incident PAD events. Factors associated with CLTI were evaluated using Cox proportional hazards regression models. Multivariate adjustment was performed for factors with *P*-values < 0.10 in the univariate analysis, including frailty score, age, BMI, education levels, smoking, DM, hypertension, hyperlipidemia, CAD, CVA or ICH, atrial fibrillation, Kt/V, hemoglobin, antiplatelet, and statin. The proportionality assumption was graphically checked using a log-log plot and was found to be acceptable for the selected factors. All *P*-values were two-tailed, and statistical significance was set at *P* < 0.05. Statistical analysis was performed using the R language and RStudio version 4.2.2, and the gtsummary package was used.

### Data availability statement

The data that support the findings of this study are not publicly available but are available from the corresponding authors (CCW and CWY) upon reasonable request.

## Supplementary Materials

Supplementary Figure 1

Supplementary Tables
