# Supplementary material for: Association of frailty and chronic limb-threatening ischemia in patients on maintenance hemodialysis: a prospective cohort study
Source: Aging (Albany NY). 2024 Dec 31;16(22):13676–92. doi: 10.18632/aging.206178 (PMC11723663; doi:10.18632/aging.206178)
Supplement: Supplementary Figure 1 [file aging-16-206178-s001.pdf]

SUPPLEMENTARY FIGURE

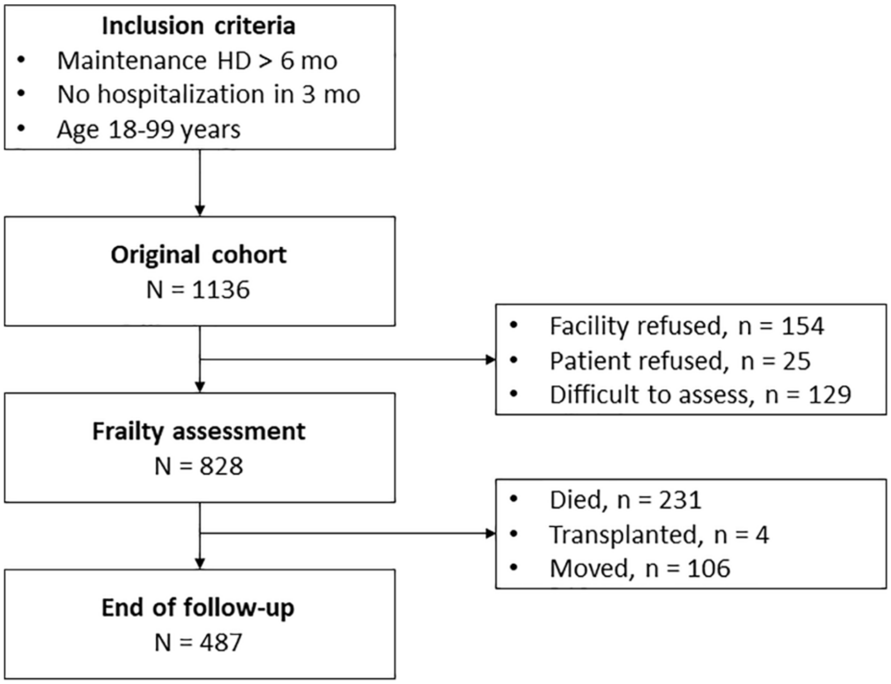

Supplementary Figure 1. Flow diagram of the study participants. Abbreviations: HD: hemodialysis; mo: months.
