# Supplementary material for: Association of frailty and chronic limb-threatening ischemia in patients on maintenance hemodialysis: a prospective cohort study
Source: Aging (Albany NY). 2024 Dec 31;16(22):13676–92. doi: 10.18632/aging.206178 (PMC11723663; doi:10.18632/aging.206178)
Supplement: Supplementary Tables [file aging-16-206178-s002.pdf]

SUPPLEMENTARY TABLES

Supplementary Table 1. Comparison of the characteristics of the study cohort and the 2017 nationwide registry in Taiwan.

Factors	Study cohort *N* = 828	Nationwide registry *N* = 82031
Age, years	67	67
Male sex (%)	52%	52%
Diabetes (%)	53%	45%
Hypertension (%)	87%	84%
Coronary artery disease (%)	26%	37%
Heart failure (%)	12%	15%
Atrial fibrillation (%)	14%	6%
Cerebrovascular disease (%)	9%	12%
Hospital-based center (%)	55%	62%
Albumin, <3.5 g/L	12%	18%
Hemoglobin, <10 g/dL	24%	40%

Supplementary Table 2. Logistic regression analysis of variables associated with frailty.

Characteristic	Univariable			Multivariable		
	OR[1]	95% CI[1]	*P*-value	OR[1]	95% CI[1]	*P*-value
**Demographic factors**						
Age >65 years	3.01	2.22–4.10	<0.001	2.16	1.51–3.10	<0.001
Female sex	1.59	1.19–2.13	0.002	1.41	1.03–1.94	0.03
**BMI groups (kg/m^2)**						
<18.5	—	—				
18.5–24	0.70	0.45–1.10	0.11			
24–27	0.71	0.43–1.19	0.19			
>27	0.63	0.36–1.10	0.10			
**Socioeconomic factors**						
Education level <6 years	2.05	1.53–2.76	<0.001	1.20	0.85–1.69	0.30
Married	0.76	0.55–1.07	0.11			
Current smoker	0.75	0.49–1.14	0.19			
**Comorbidities**						
DM	2.10	1.56–2.83	<0.001	1.77	1.25–2.54	0.002
HTN	0.62	0.41–0.92	0.02	0.81	0.51–1.29	0.40
Hyperlipidemia	0.78	0.55–1.10	0.16			
CAD	1.41	1.02–1.94	0.04	1.14	0.80–1.62	0.50
CVA or ICH	2.11	1.31–3.39	0.002	1.80	1.09–2.99	0.02
CHF	1.33	0.86–2.03	0.19			
Atrial fibrillation	1.66	1.11–2.48	0.01	1.52	0.99–2.34	0.06
COPD	0.51	0.15–1.43	0.24			
**Dialysis factors**						
Dialysis vintage (years)	0.97	0.95–1.00	0.07	1.00	0.97–1.04	0.80

Cholesterol (mg/dL)	1.00	0.99–1.00	0.43			
Albumin (g/dL)	0.30	0.19–0.47	<0.001	0.49	0.29–0.82	0.01
Kt/V (Daugirdas)	0.94	0.57–1.53	0.80			
Hb (g/dL)	0.88	0.79–0.98	0.02	0.95	0.84–1.08	0.50

[1]OR: Odds Ratio; CI: Confidence Interval. Abbreviations: BMI: body mass index; CAD: coronary artery disease; CHF: congestive heart failure; COPD: chronic obstructive pulmonary disease; CVA: cerebrovascular accident; DM: diabetes mellitus; Hb: hemoglobin; HTN: hypertension; ICH: intracerebral hemorrhage.

Supplementary Table 3. Mortality and major adverse limb events.

	**Overall, *N* = 828**	**Not Frail, *N* = 231**	**Pre-Frail, *N* = 317**	**Frail, *N* = 280**
CLTI	104 (13%)	15 (6.5%)	34 (11%)	55 (20%)
Amputation	28 (3%)	2 (1%)	10 (3%)	16 (6%)
Interventions	101 (12%)	15 (7%)	35 (11%)	51 (18%)
MALE	108 (13%)	16 (7%)	40 (13%)	52 (19%)
Mortality	231 (28%)	31 (13%)	85 (27%)	115 (41%)

Interventions: angioplasty or surgery for peripheral arterial disease. Abbreviations: CLTI: chronic limb-threatening ischemia; MALE: major adverse limb events: a combination of amputation and interventions.
